# Impact of prerelease methadone on mortality among people with HIV and opioid use disorder after prison release: results from a randomized and participant choice open-label trial in Malaysia

**DOI:** 10.1186/s12879-022-07804-6

**Published:** 2022-11-11

**Authors:** Alexander R. Bazazi, Gabriel J. Culbert, Martin P. Wegman, Robert Heimer, Adeeba Kamarulzaman, Frederick L. Altice

**Affiliations:** 1 Department of Medicine, Section of Infectious Diseases, AIDS Program, Yale School of Medicine, 135 College Street, Suite 323, New Haven, CT 06510-228 USA; 2 Department of Epidemiology of Microbial Diseases, Yale School of Public Health, New Haven, CT USA; 3 Department of Psychiatry, University of California, San Francisco, San Francisco, CA USA; 4 Population Health Nursing Science, University of Illinois at Chicago, Chicago, IL USA; 5 Faculty of Medicine, Centre of Excellence for Research in AIDS (CERiA), University of Malaya, Kuala Lumpur, Malaysia

**Keywords:** Methadone, HIV, Opioid use disorder, Prison, Mortality

## Abstract

**Introduction:**

Mortality is elevated after prison release and may be higher in people with HIV and opioid use disorder (OUD). Maintenance with opioid agonist therapy (OAT) like methadone or buprenorphine reduces mortality in people with OUD and may confer benefits to people with OUD and HIV leaving prison. Survival benefits of OAT, however, have not been evaluated prospectively in people with OUD and HIV leaving prison.

**Methods:**

This study prospectively evaluated mortality after prison release and whether methadone initiated before release increased survival after release in a sample of men with HIV and OUD (n = 291). We linked national death records to data from a controlled trial of prerelease methadone initiation conducted from 2010 to 2014 with men with HIV and OUD imprisoned in Malaysia. Vital statistics were collected through 2015. Allocation to prerelease methadone was by randomization (n = 64) and participant choice (n = 246). Cox proportional hazards models were used to estimate treatment effects of prerelease methadone on postrelease survival.

**Results:**

Overall, 62 deaths occurred over 872.5 person-years (PY) of postrelease follow-up, a crude mortality rate of 71.1 deaths per 1000 PY (95% confidence interval [CI] 54.5–89.4). Most deaths were of infectious etiology, mostly related to HIV. In a modified intention-to-treat analysis, the impact of prerelease methadone on postrelease mortality was consistent with a null effect in unadjusted (hazard ratio [HR] 1.3, 95% CI 0.6–3.1) and covariate-adjusted (HR 1.2, 95% CI 0.5–2.8) models. Predictors of mortality were educational level (HR 1.4, 95% CI 1.0–1.8), pre-incarceration alcohol use (HR 2.0, 95% CI 1.1–3.9), and lower CD4^+^ T-lymphocyte count (HR 0.8 per 100-cell/mL increase, 95% CI 0.7–1.0).

**Conclusions:**

Postrelease mortality in this sample of men with HIV and OUD was extraordinarily high, and most deaths were likely of infectious etiology. No effect of prerelease methadone on postrelease mortality was observed, which may be due to study limitations or an epidemiological context in which inadequately treated HIV, and not inadequately treated OUD, is the main cause of death after prison release.

*Trial registration*: NCT02396979. Retrospectively registered 24/03/2015

**Supplementary Information:**

The online version contains supplementary material available at 10.1186/s12879-022-07804-6.

## Introduction

Opioids are responsible for 70% of the years of healthy life lost annually to substance use [1]. In 2016, an estimated 26.8 million people worldwide were living with an opioid use disorder (OUD) [2]. Injection of opioids with contaminated equipment remains a key driver of the HIV/AIDS pandemic in many regions [3]. Punitive drug policies have resulted in high rates of incarceration among people with OUD, many of whom are people with HIV (PWH). An estimated 3.8% of people in prison worldwide are HIV-positive [4], and one third to one half of the global prison population are people who inject drugs [5]. Given the high prevalence of HIV and OUD in prison populations, the provision of humane and evidence-based HIV and substance use disorder treatment in prisons is both a human rights and public health imperative [6]. In many countries, however, prisons are not equipped to provide such services [7]. Nor do many coordinate transitional services for postrelease continuity of care. Consequently, discontinuation of treatment, relapse to substance use, and viral rebound often occur in people with OUD and HIV following prison release [8]. This combination of significant treatment needs and structural barriers to care before and after prison release contributes to the overall high global mortality in people released from correctional facilities [9, 10].

In addition to the health risks many people encounter when they leave prison, people with OUD and HIV face risks related to these conditions. Drug overdose is the most common preventable cause of death in people with OUD and in people released from prison worldwide [9, 11]. Even after long periods of abstinence during incarceration, relapse to opioid use after prison release approaches 90% among those with OUD and contributes substantially to mortality that occurs during the immediate postrelease period [10, 12–14]. The share of postrelease deaths attributable to HIV in the U.S. and other high-income countries has declined [15]. Linkage to care postrelease, however, remains suboptimal [16], and even in the U.S., HIV may supersede drug overdose as the leading cause of postrelease death in people with HIV [17]. Studies of postrelease mortality in middle-income countries, where HIV prevalence in prisons is higher, have concluded similarly that people with HIV are at substantial risk for HIV-related opportunistic infections and HIV-related death after prison release [18, 19]. This study was conducted in Malaysia, where HIV prevalence is significantly higher among men in prison compared with men in the general population, and most incarcerated men with HIV also meet criteria for OUD [20, 21]. Given the high burden of medical and psychiatric comorbidities in this population, mortality risk may be high [20–23].

Maintenance with opioid agonist therapies (OAT) like methadone or buprenorphine are the most effective strategies for treating OUD [24]. Evidence shows that OAT reduces non-prescribed opioid use, overdose, mortality, and injection-related HIV risk behaviors and improves health and social functioning [25–29]. Moreover, OAT that is started within prison increases linkage and retention to OAT in the community postrelease [30–38]. Two large retrospective cohort studies from Australia and England showed that prerelease OAT was associated with lower all-cause mortality in the four weeks postrelease [14, 39]. Additionally, OAT improves HIV-related health outcomes, including initiation of antiretroviral therapy (ART), ART adherence, and viral suppression, which is the single-largest contributor to decreasing HIV-related mortality [40].

In this study, we estimated the effect of prerelease methadone initiation on postrelease mortality in men with HIV and OUD. Our hypothesis, based on formative work in Malaysia [41, 42] and studies of prerelease methadone in Kyrgyzstan [43] and the U.S. [32], was that starting methadone within prison would reduce postrelease mortality by decreasing opioid overdose and increasing engagement in HIV care and adherence to ART. To test this hypothesis, we linked national death records to data from an open-label trial of prerelease methadone conducted with imprisoned and soon-to-be-released men with OUD and HIV in Malaysia. This study is, to our knowledge, the first to examine postrelease mortality and the possible survival benefits of prerelease OAT in this population.

## Methods

### Study design

Project *Harapan* was designed as a 2 × 2 factorial randomized controlled trial of (a) prerelease methadone and (b) an evidence-based group behavioral intervention, the Holistic Health Recovery Program (HHRP) [44, 45]. This trial has been described previously [44] and is registered at ClinicalTrials.gov (NCT02396979). Briefly, men with HIV and OUD were recruited from Malaysia’s largest all-male prison and allocated to receive prerelease methadone, initially by randomization and later by participant choice. Participants allocated to prerelease methadone were started on daily methadone before their release date with planned linkage to government-subsidized methadone in the community postrelease. Allocation to the behavioral intervention, HHRP, was by randomization throughout the study. Participants allocated to HHRP participated in eight group sessions led by a study counselor using the adapted HHRP curriculum. Standard care for all participants included written information about community HIV and substance use disorder treatment services upon release.

The primary outcome of the trial was sexual and injection-related risk behaviors during the first year postrelease [44, 46]. In this paper, we present a secondary analysis of post-release mortality that was developed to more closely examine high rates of study attrition amidst high numbers of participant deaths. To analyze survival in this cohort, we linked death certificate information from Malaysia’s national vital statistics registry to biological and survey data collected from participants at the time of enrollment, one to six months before their anticipated prison release date. These baseline data included whether they were allocated to receive prerelease methadone, either by randomization or choice. The study’s time-limited behavioral intervention, HHRP, was not theorized to influence mortality but was included in this analysis for completeness.

### Recruitment and eligibility

HIV testing in Malaysia’s prisons is compulsory. Participants were recruited from a segregated housing unit for HIV-diagnosed men in Malaysia’s largest all-male prison located in Greater Kuala Lumpur. Men from this housing unit attended voluntary group meetings where researchers provided information about the study. Men who expressed interest in the study were given an appointment to meet with researchers privately to assess for eligibility and engage in the informed consent process. Eligible participants were Malaysian citizens 18 years or older, HIV seropositive (confirmed at enrollment), meeting DSM-IV criteria for opioid dependence in the 12 months prior to incarceration, within 6 months of their prison release date, and planned residence within the study’s catchment area (Greater Kuala Lumpur) after release. Enrollment and all follow-up visits occurred between 2010 and 2014. Target sample size was 400 participants based on calculations for the original fully randomized design, which was meant to achieve 85% power to detect a 10% difference in the rate of HIV transmission behaviors [44].

### Allocation to prerelease methadone

All participants were screened and tested negative for opioids at enrollment. The first 63 participants were recruited into the fully randomized 2 × 2 design, with assignment to methadone by random allocation sequence with blocking, administered by one study staff member not involved in recruitment. After 63 participants were recruited into the fully randomized 2 × 2 design, the assignment mechanism for methadone was changed from randomization to participant choice. This change to offer participants a choice as to whether they would receive prerelease methadone was necessitated by two key developments. First, study enrollment was initially slow due to strong individual preferences for and against methadone with individuals declining enrollment due to concern that they would not receive their preferred treatment [41, 42]. Second, the Ministry of Health liberalized access to methadone in prisons due to human rights concerns from withholding evidenced-based treatment for OUD and waived restrictions including a requirement for families to consent. Subsequently, protocols were amended to allow allocation to methadone based on participant preference. After explaining the possible risks and benefits of treatment with methadone, researchers asked participants to choose whether or not to participate in the study’s prerelease methadone induction and maintenance protocols. Medical records were queried to ascertain methadone dose at the time of release.

### Study interventions

#### Methadone initiation within prison and linkage to subsidized methadone postrelease

Protocols for methadone induction called for participants to be started on 5 mg daily from one to six months before prison release, which was increased by 5 mg weekly to target a daily dose of at least 80 mg. In addition, participants allocated to prerelease methadone were referred and transported by research staff, whenever possible, to a fully subsidized methadone treatment program in the community after release. Standard care for all participants included written information about community HIV and drug treatment services, including government-subsidized methadone treatment programs.

#### Holistic Health Recovery Program (HHRP) behavioral intervention

HHRP is an evidence-based behavioral intervention designed to prevent HIV transmission, increase adherence to ART, and strengthen drug relapse prevention skills [47–49]. HHRP was previously adapted for the Malaysian prison and postrelease settings [44, 45]. Participants randomized to HHRP participated in eight two-hour group sessions before prison release led by study staff followed by an optional “booster session” 1 month after release.

### Mortality ascertainment

**Fig. 1:**
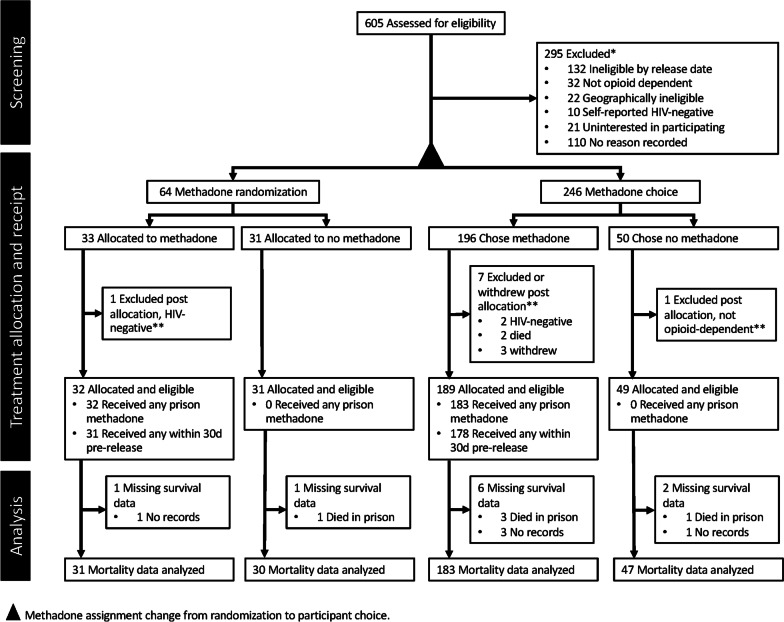
Participant flow diagram. *Reasons for exclusion not mutually exclusive. **All post randomization withdrawals and deaths occurred shortly after screening and before intervention receipt

The period of observation for survival was the interval between the date that the first participant was released from prison (8/2010) and 22 months after the last participant was released from prison (10/2015), when the vital statistics registry was queried for the last time. Data queries submitted to the Ministry of Health included the participant’s name, date of birth, and government-issued identification number. These identifiers were matched to vital records and returned with an indicator for whether the individual was alive or deceased. Records for decedents included the date and cause of death as submitted by the pronouncing physician. For this study, we categorized causes of death as either infectious (e.g., tuberculosis, sepsis) or non-infectious (e.g., cardiovascular disease, trauma). Participants who were matched to vital records but had no record of death were considered alive and censored on the date their records were matched. Twelve participants could not be matched with vital records. Of these, seven were matched with study records showing dates of attendance at postrelease clinic or study visits or dates of rearrest, which were used for censoring. The other five participants without matching vital records had no record of postrelease contact or rearrest and were effectively censored on their release date and excluded from analysis. Also excluded were five participants who died before release from the index incarceration, leaving 291 participants in the final analytic sample (Fig. 1). In a sensitivity analysis, we examined whether excluding these ten participants may have introduced bias.

### Statistical methods

#### Approach to the change in allocation mechanism for methadone

Data from participants in the randomization and treatment choice phases were pooled to maximize power to detect a methadone treatment effect in the primary analysis. Separate analyses of participants allocated to methadone by randomization versus choice are presented in Additional file 1. When estimating treatment effects in the pooled sample, we also included terms for treatment phase (randomization vs. choice) and its interaction with treatment, which allowed the effect of methadone to vary between individuals in the randomization and choice phases independently of differences in baseline characteristics. We performed two “as-treated” analyses, defining prerelease methadone treatment as: (1) receipt of at least one dose of methadone in the 30 days before release, and (2) receipt of at least a 60 mg daily methadone dose prior to release, based on evidence that doses > 60 mg are more effective [50].

#### Survival analysis

Cox proportional hazards models with robust standard errors were used to estimate treatment effects [51] in three models. Model 1 included terms for methadone, treatment phase, and their interaction. Model 2 added baseline CD4 + T-cell count, which we considered a priori to be an important determinant of mortality. Model 3 included 15 additional baseline covariates, selected a priori as theoretical predictors of postrelease mortality or confounders of the relationship between methadone and mortality. We present equivalent models separating the data for participants in the randomization and treatment choice phases separately [see Additional file 1]. We evaluated evidence of deviation from the proportional hazards assumption by testing for independence of the Schoenfeld’s residuals and time. Descriptions of baseline covariates are presented in Additional file 1 [52, 53].

All primary analyses were by modified intention-to-treat, with exclusions as defined above. As-treated analyses were also performed using the same set of models except with treatment defined as: (1) receiving at least one methadone dose in the 30 days before release, and (2) receiving at least 60 mg of methadone before release. As a sensitivity analysis, we also analyzed survival allowing participants to contribute a maximum of 12 months of survival time to prevent those who participated earlier in the trial from contributing disproportionality more person-years of observation.

We present Kaplan–Meier survival curves as well as survival curves adjusted for baseline covariates with inverse probability weighting [54, 55]. Weights were estimated with logistic regression and stabilized using the marginal probability of receiving methadone.

#### Mortality rate estimation

Postrelease crude mortality rates (CMRs) are presented with standard errors estimated using the nonparametric bootstrap, resampling within study phase. The standardized mortality ratio (SMR) was estimated using life tables from the World Health Organization, standardizing by age, gender, and year in Malaysia, in the R package *popEpi* with standard errors from exact Poisson intervals [56]. As a sensitivity analysis, we also estimated within-prison CMRs using the analytic sample described above of 291 plus the five participants who died within prison.

#### Missing data

Regression analysis showed no difference in baseline characteristics of participants included in the analysis (n = 291) and those excluded (n = 10) because they died within prison or were missing from the vital registry and had no postrelease study contact (see Additional file 1). We imputed median values for eight participants missing responses for one or more baseline covariates. Unadjusted treatment effect estimates were unaffected by this imputation. Models adjusted only for CD4^+^ T-cell count have values imputed for two participants.

### Ethical considerations

Informed consent was obtained from all study participants prior to enrollment and was repeated after prison release. Participants consented to collection of medical and administrative records in advance. This study was approved by institutional review boards at Yale University, University Malaya Medical Centre, and the Office of Human Research Protection at the U.S. Department of Health and Human Services.

## Results

### Participant flow

A study flow diagram is shown in Fig. 1. From 291 participants included in this analytic sample, 214 (78.7%) were allocated to prerelease methadone, which includes 54.1% (33/61) of those enrolled in the randomization phase and 79.6% (183/230) of those enrolled in the treatment choice phase. Most participants allocated to prerelease methadone (203/214, 94.8%) received at least one dose of methadone in the 30 days before release. Two thirds of participants allocated to methadone (121 of the 184 for whom complete data were available) were receiving a daily methadone dose of at least 60 mg before release.

### Baseline characteristics

Participant characteristics are shown in Table 1. Male participants were 39 years of age on average, serving an average prison sentence of less than 3 years (mean 33.4 months). All participants were opioid dependent, per study eligibility, and nearly all participants (95.2%) reported a history of injection drug use. CD4^+^ T-lymphocyte testing at enrollment found one in six (16.2%) to be severely immunocompromised (< 200 CD4^+^ T-lymphocyte cells/µL). Yet, only one in five (20.3%) reported having ever received ART in the community or prison at the time of study enrollment.

**Table 1:** Baseline characteristics of study participants

	Overall (n = 291)	Methadone (n = 214)	No methadone (n = 77)	p-value
Age (years)	39.0 (SD 6.6)	38.7 (SD 6.5)	39.9 (SD 6.7)	0.290
Malay ethnicity	212 (72.9%)	159 (74.3%)	53 (68.8%)	0.372
Married	32 (11.0%)	26 (12.1%)	6 (7.8%)	0.254
Educational attainment	2.29 (SD 1.05)	2.27 (SD 1.1)	2.33 (SD 1.0)	0.632
Employed^†^	186 (63.9%)	141 (65.9%)	45 (58.4%)	0.259
CD4+ T-cell count (cells/mm^3^)	445 (SD 282)	432 (SD 274)	482 (SD 303)	0.270
HIV symptom index (0–20)	4.9 (SD 3.6)	4.8 (SD 3.4)	5.1 (SD 4.1)	0.824
History of tuberculosis	67 (23.0%)	48 (22.4%)	19 (24.7%)	0.695
Addiction severity index (0–100)^§^	24.5 (SD 9.6)	24.5 (SD 9.4)	28.0 (SD 9.5)	0.002
Ever injected drugs	277 (95.2%)	202 (94.4%)	75 (97.4%)	0.213
Benzodiazepine use^†^	34 (11.7%)	21 (9.8%)	13 (16.9%)	0.140
Methamphetamine use^†^	161 (55.3%)	121 (56.5%)	40 (51.9%)	0.492
Opioid use^†^	271 (93.1%)	198 (92.5%)	73 (94.8%)	0.466
Alcohol use^†^	56 (19.2%)	41 (19.2%)	15 (19.5%)	0.952

Data are mean (SD) or n (%). P-values are from t-tests of difference in means for dichotomous variables and KS tests of difference in distributions for continuous variables. ^†^Assessed in 30-day period before incarceration. ^§^Addiction severity index drug composite score

Few participants reported a history of maintenance treatment with methadone (9.6%), though 31.6% had ever used methadone and 17.5% had used methadone in the 30 days prior to incarceration. Addiction severity was higher among those not allocated to prerelease methadone versus those allocated to receive methadone (p = 0.002). A joint test of overall difference between methadone versus no methadone participants on all baseline covariates in Table 1 was not significant (p = 0.163, F-test). Baseline differences between participants in the randomization and treatment choice phase are shown in Additional file 1.

### Mortality estimates

Matching death records for 62 participants were returned from the national registry which occurred over 872.5 person-years of postrelease observation. Deaths occurred as early as 23 days after release and almost one third of all deaths (19/62) occurred within the first year postrelease. Most deaths (53/62) were judged likely to be of infectious etiology (see Additional file 1).

As shown in Table 2, the postrelease crude mortality rate (CMR) was 71.1 deaths per 1000 person-years (PY, 95% CI 54.5–89.3). Standardized mortality ratio (SMR) estimates showed that, after leaving prison, men in our sample died at 20 times the rate (SMR 20.1, 95% CI 15.4–25.8) of men the same age in the general population in Malaysia. Results were similar when using data only from participants’ first year postrelease (SMR 20.2, 95% CI 12.2–31.6). CMR estimates were similar for those allocated to methadone versus those who were not (Table 2). Mortality rates were higher in the initial randomization phase of the study than in the subsequent treatment choice phase (see Additional file 1).

**Table 2:** Crude mortality rates after prison release stratified by prerelease methadone allocation

	Time (PY)	Deaths	Deaths per 1000 PY (95% CI)
Methadone	607.9	43	70.7 (50.8–93.4)
No methadone	264.6	19	71.8 (43.4–105.4)
Overall	872.5^†^	62	71.1 (54.5–89.4)

PY: person years; CI: confidence interval. ^†^Mean PY of observation 3.0, range 0.06–6.3, SD 4.4

The estimated CMR within prison (65.1 deaths per 1000 PY, 95% CI 21.1–151.9) was broadly overlapping with the postrelease CMR estimate. Comparison of within-prison mortality between those allocated to methadone (54.6 per 1000 PY, 95% CI 11.3–159.6) and those not allocated to methadone (91.2 per 1000 PY, 95% CI 11.0–329.5) are limited by imprecision of the estimates due to limited observations.

### Survival analysis

**Fig. 2:**
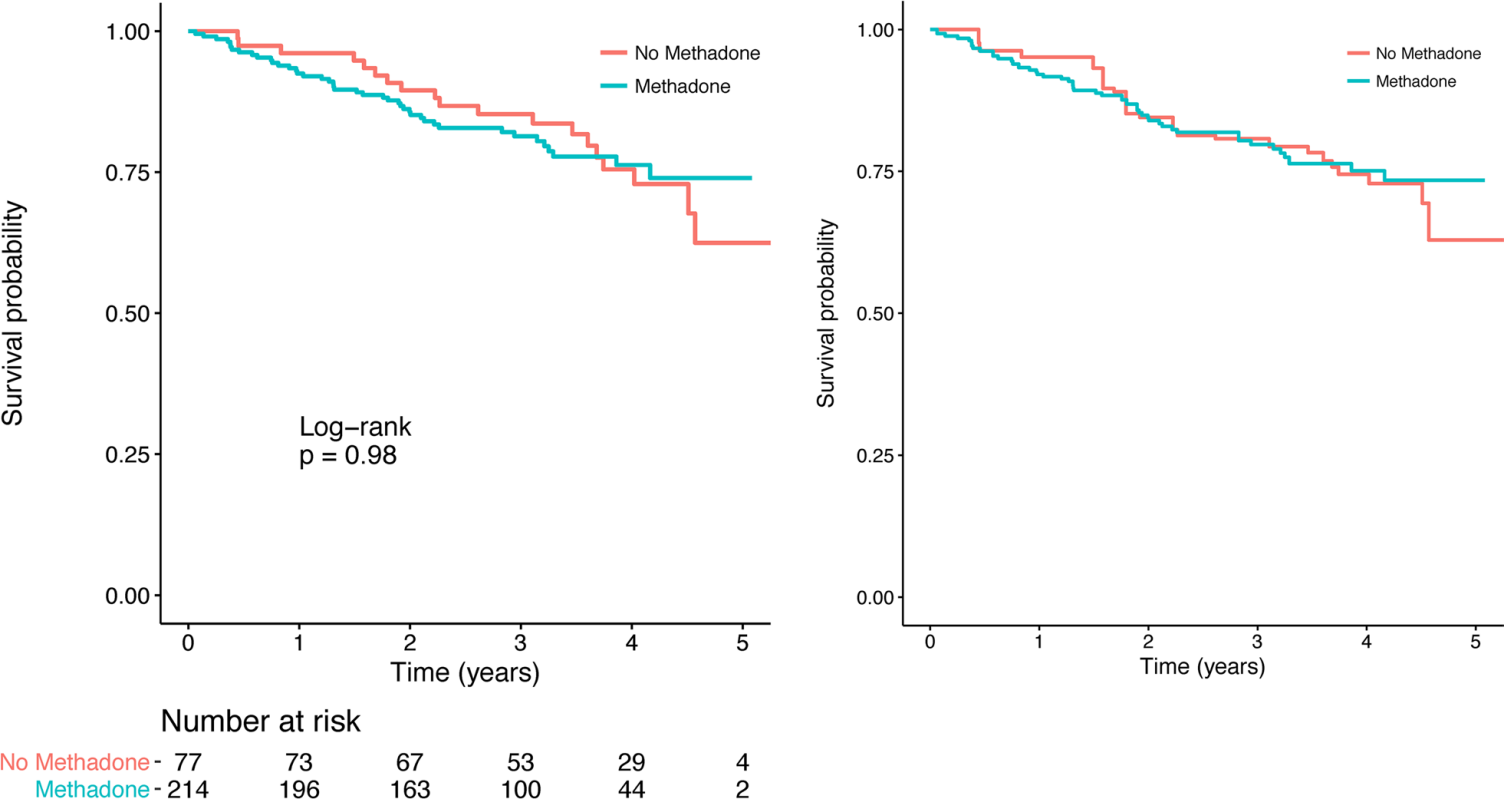
Unadjusted and adjusted probability of survival. Left panel showing unadjusted probability of survival, Kaplan–Meier survival curves. Right panel showing Kaplan–Meier survival curves adjusted for baseline covariates using inverse probability weighting

Unadjusted and adjusted survival curves for participants allocated to methadone versus no methadone are shown in Fig. 2. Cox proportional hazards models are shown in Table 3. Estimates from each of the three analyses were consistent with a null effect of allocation to prerelease methadone on survival after prison release. In the most basic model, the hazard ratio (HR) for methadone was 1.3 (95% CI 0.6–3.0). After adjusting for baseline CD4^+^ T-cell count, the HR for methadone was 1.2 (95% CI 0.5–2.8). Adjusting for the full covariate set, the HR was 1.2 (95% CI 0.5–2.8; Table 3). In the full model, higher baseline CD4^+^ T-cell count was protective (HR 0.8 for each 100-cell/mL increase, 95% CI 0.72–0.97). Alcohol use in the 30 days prior to incarceration was associated with decreased survival (HR 2.0, 95% CI 1.1–3.9).

**Table 3:** Cox proportional hazards models of mortality after prison release

	Model 1 HR (95% CI)	Model 2 HR (95% CI)	Model 3 HR (95% CI)
Methadone	1.33 (0.58–3.05)	1.22 (0.53–2.80)	1.18 (0.49–2.83)
Randomized allocation phase	1.99 (0.82–4.84)	1.84 (0.76–4.46)	1.65 (0.58–4.67)
Methadone*allocation phase	0.69 (0.21–2.23)	0.70 (0.22–2.24)	0.66 (0.19–2.32)
HHRP	1.34 (0.81–2.22)	1.30 (0.78–2.17)	0.99 (0.56–1.76)
Age (years)			0.98 (0.93–1.04)
Malay ethnicity			0.61 (0.29–1.29)
Married			1.97 (0.92–4.21)
Educational attainment			1.35 (1.04–1.76)
Employed^†^			0.59 (0.33–1.04)
CD4+ T-cell count^‡^		0.90 (0.82–1.00)	0.84 (0.72–0.97)
HIV symptom index			0.97 (0.90–1.05)
History of tuberculosis			0.55 (0.24–1.27)
Addiction severity index^§^			0.98 (0.95–1.02)
History of drug injection			1.05 (0.38–2.90)
Benzodiazepine use^†^			1.82 (0.81–4.13)
Methamphetamine use^†^			0.83 (0.42–1.62)
Opioid use^†^			2.50 (0.56–11.1)
Alcohol use^†^			2.02 (1.06–3.86)

Randomized allocation phase is an indicator variable for allocation to methadone by randomization versus participant choice. HHRP: Holistic Health and Recovery Program; HR: hazard ratio; CI: confidence interval. ^†^Assessed in the 30 days before incarceration. ^‡^Represents 100-cell/mm^3^ change. ^§^Addiction severity index drug composite score

Survival models, fit separately with participants allocated to prerelease methadone in the randomization phase and those allocated in the participant choice phase, yielded similar estimates consistent with a null effect of prerelease methadone (see Additional file 1). As-treated analyses defining treatment as both receipt of any methadone before release and as receipt of at least 60 mg of methadone before release also yielded estimates consistent with a null effect (see Additional file 1).

#### Additional sensitivity analyses and robustness checks

We performed two sensitivity analyses to examine whether the effects of methadone on survival varied over time. In the first of these analyses, survival time was truncated to allow each participant to contribute a maximum of one person-year of observation, regardless of when they were released. The second analysis included a time-dependent variable to account for any changes in methadone’s effects that may have occurred during the study period. Neither analysis showed evidence of an association between allocation to prerelease methadone and survival. No evidence was found to suggest that the proportional hazards assumption was violated for the intervention effect (data not shown). A joint test comparing the ten excluded participants with the analytic sample (n = 291) on baseline characteristics was not significant, suggesting that exclusion of these participants was unlikely to bias our treatment effect estimates (see Additional file 1).

## Discussion

The postrelease mortality rate was extraordinarily high (71.1 deaths per 1000 PY) in this sample of men with OUD and HIV. Although few studies provide an opportunity for direct comparison, studies in populations with one or more of these risk factors have documented far lower mortality rates. For comparison, mortality among released prisoners globally is estimated at about 10 deaths per 1000 person-years [9, 57]. Mortality rates lower than ours also have been observed in people with HIV who use opioids (28.6 deaths per 1000 PY), released prisoners with untreated OUD in Australia (36.7 deaths per 1000 PY) [14] and Taiwan (26 deaths per 1000 PY) [58], and even in released prisoners with HIV in French Guiana (33.8 deaths per 1000 PY) [18] and the U.S. (28.6 deaths per 1000 PY) [59]. Only one study, a cohort of men with HIV released from prisons in Indonesia, documented higher mortality rates (215 deaths per 1000 PY), but was based on a relatively small sample [19]. Elevated mortality rates in our study and the Indonesian cohort are unsurprising, perhaps, given the combined risk of OUD, HIV, and prison release in these cohorts.

The few existing studies of prerelease methadone that have examined postrelease mortality have generally shown some survival benefit, which stands in contrast to our study that failed to detect a postrelease survival benefit. Two large cohort studies in Australia and England, both with very little PWH, concluded that prerelease OAT increased survival, but only in the first postrelease month [14, 39]. A three-arm randomized trial in the US showed one postrelease death in each of the two groups treated with methadone (immediately before or after prison release) compared to six deaths in a group that received neither [32]. In one other trial in the U.S. examining within-prison methadone, postrelease mortality was negligible [31]. The hypothesized pathway through which within-prison methadone would reduce postrelease mortality is through increasing postrelease methadone treatment, though incomplete data limited our ability to conduct an as-treated analysis accounting for post-release methadone receipt.

Our study differs from prior research on prerelease methadone and postrelease mortality in that it included only people with HIV. This difference in inclusion criteria alongside critical differences in social and epidemiological context in Malaysia versus higher-income settings may explain in part why we did not detect any postrelease survival benefit from prerelease methadone. The benefits of prerelease methadone have mainly been demonstrated in high-income countries where life expectancy for people with HIV is higher and OAT effectively prevents relapse to opioids and overdose [30–34], which is the leading cause of postrelease mortality in these settings [9]. By contrast, our sample was drawn from a prison population with numerous HIV-related health risks and perhaps fewer risks related to opioid use. In Malaysia, while non-fatal overdose among people who use opioids is common [23], rates of fatal overdose are unknown, as are the relative contributions of HIV and opioid-related mortality in this population. For men with OUD and HIV released from prison in Malaysia, the risk of death associated with advanced HIV disease may supersede the risks associated with opioid use disorder, treated or not.

We cannot exclude overdose as a cause of death due to possible underreporting, yet no cases of fatal overdose were reported in our data. Our findings are consistent, however, with the theory that inadequately treated HIV, not overdose, is the primary contributor to postrelease mortality in this population. Moreover, given that most of the participants in this study selected methadone, those at highest risk for overdose may have selected a protective treatment. As further evidence, baseline CD4^+^ T-lymphocyte count strongly predicted mortality, and most causes of death recorded by pronouncing physicians identified some underlying infectious etiology, many of which represented AIDS-defining illnesses. Thus, in incarcerated persons leaving prison, untreated HIV may serve as a more proximal risk for death. Finally, we did not observe markedly higher mortality rates in the period immediately after release, as would be expected if deaths were caused by opioid overdose [10].

While evidence from other settings has shown that OAT can improve HIV treatment outcomes [40], this may not have occurred in our sample. Reasons may include that despite initiating methadone in prison, participants may not have adhered to it or may have discontinued it postrelease. Additionally, linkage to HIV care postrelease may have been poor due to structural barriers. In some high-income settings, postrelease linkage to ART has been found to be low [60], and this is also likely to be the case in Malaysia, where the number of people with HIV receiving treatment is low despite ART being fully subsidized [61]. Provider discrimination may play a role: more than half of sampled Malaysian HIV care providers in one study were unwilling to start ART in patients who injected drugs or were recently released from prison, even at very low CD4^+^ T-cell counts [62, 63]. In addition to stigma and discrimination, the intersection of poorly treated HIV and other medical and social vulnerabilities, such as untreated tuberculosis or chronic diseases, lack of housing, and food insecurity, may increase risk of death. Improving access to quality care for HIV and other medical comorbidities as well as social services for people who use drugs in Malaysia will be a crucial step to reducing health disparities in this population [62].

Factors related to methadone implementation also may explain why we failed to detect an impact of prerelease methadone on postrelease mortality in this study. First, participants allocated to methadone may not have reached an optimal dose before release for reasons including inability to tolerate the titration schedule or being released early before completion of the titration [42]. An as-treated analysis defining treatment as receipt of at least 60 mg of methadone prerelease, however, also was consistent with a null effect; other studies have suggested that optimal doses to engage persons released from prison should receive higher dosages in excess of 80mg [42, 43]. Second, some participants allocated to receive methadone may not have been linked successfully to methadone treatment after release, and some participants not allocated to receive prerelease methadone may have initiated community methadone treatment postrelease. A main study limitation is the absence of complete data on methadone utilization postrelease. Fourth, because the study was not powered to detect survival differences, absence of evidence for a treatment effect could be due to low power. Fifth, selection bias among non-randomized participants due to factors omitted in our adjustment strategy could have biased our estimates toward a null treatment effect. We did not, however, detect an effect in the subsample of randomized participants, where selection bias was not an issue, though our power to detect an effect in this subsample was lower. Finally, we were unable to distinguish deaths that occurred in the community from deaths that may have occurred in prison among participants who were reincarcerated during the postrelease observation period. It should be noted that methadone could have positively impacted other health and social outcomes in our sample including potentially by reducing injection drug use, but this was not the focus of this analysis. Implementation challenges as well as barriers and facilitators to postrelease retention in this trial have been previously reported [44, 46].

Of interest, pre-incarceration alcohol use, reported by 19.2% of participants, was predictive of postrelease mortality. In people with HIV, alcohol use is associated with increased mortality [64]. Alcohol use also has been implicated in overdose deaths in people who use opioids [23, 65], and in the deaths of people receiving methadone for opioid use disorder [66, 67]. One promising intervention meriting further investigation is extended-release naltrexone, which has been associated with improved postrelease HIV treatment outcomes separately in people with opioid and alcohol use disorders [68, 69].

## Conclusions

In men with HIV and opioid use disorder in Malaysia, mortality after prison release is high and mainly attributable to HIV/AIDS. Prerelease methadone that has been shown elsewhere to improve health outcomes related to substance use and HIV was not associated with a mortality benefit in this study, which may be due to study limitations as well as an epidemiological context in which advanced and poorly managed HIV superseded opioid-related fatalities as the predominant driver of mortality. Improving patient access to ART and ensuring continuation of ART postrelease is urgently needed to reduce mortality among men with HIV and opioid use disorder in Malaysia. Future research should continue to explore promising biomedical interventions (including patient preferences for and use of long-acting injectable agents for OUD and HIV), behavioral interventions (including peer navigation), and structural interventions (including targeting provider discrimination, lowering barriers to treatment entry, and collocating services) to improve HIV and OUD treatment outcomes in this population.

## Supplementary Information

**Additional file 1. **Appendix. Detailed description of covariates, supplementary analyses not shown in main text, and raw data on cause of death classification.

## Data Availability

Individual-level cause of death data can be found in Additional file 1. The complete dataset used in this analysis contains highly sensitive individual-level measures, including incarceration history, illegal behaviors, HIV status, and death records, in a relatively small population of PLWH released from one correctional facility over several years, which elevates the risk to participants of publicly sharing the dataset. Additionally, the death records linked to participant trial data are regulated by the Clinical Research Center of the National Institute of Health in the Ministry of Health and the National Registration Department of the Ministry of Home Affairs. Data will be made available to researchers who receive permission from these organizations and meet criteria for access to confidential data. Interested researchers should contact contact@crc.gov.my.

## Abbreviations

CI: Confident interval
CMR: Crude mortality rate
HR: Hazard ratio
HHRP: Holistic Health Recovery Program
OAT: Opioid agonist therapies
OUD: Opioid use disorder
PWH: People with HIV
PY: Person-years
SMR: Standardized mortality ratio
