# Supplementary material for: Impact of prerelease methadone on mortality among people with HIV and opioid use disorder after prison release: results from a randomized and participant choice open-label trial in Malaysia
Source: BMC Infect Dis. 2022 Nov 11;22:837. doi: 10.1186/s12879-022-07804-6 (PMC9652918; doi:10.1186/s12879-022-07804-6)
Supplement: Supplementary file 1 — Additional file 1. Appendix. Detailed description of covariates, supplementary analyses not shown in main text, and raw data on cause of death classification. [file 12879_2022_7804_MOESM1_ESM.pdf]

Appendix

Contents

1 Description of covariates **3**

2 Testing the association between treatment group and outcome missingness **4**

3 Differences in baseline characteristics between randomized and treatment choice phase participants **6**

4 Mortality rates by study phase **7**

5 Causes of death **8**

6 Treatment effect estimation by study phase **10**

7 Sensitivity analyses **16**

8 As-treated analyses **18**

List of Figures

1 Unadjusted Kaplan-Meier survival curve of participants treated with within-prison methadone and controls in the **randomization phase.** 12
2 Adjusted Kaplan-Meier survival curve of participants treated with within-prison methadone and controls in the **randomization phase.** Curves are adjusted for baseline covariates using inverse probability weighting. 13
3 Unadjusted Kaplan-Meier survival curves of participants treated with within-prison methadone and controls in the **treatment choice phase.** 14
4 Adjusted Kaplan-Meier survival curves of participants treated with within-prison methadone and controls in the **treatment choice phase.** Curves are adjusted for baseline covariates using inverse probability weighting. 15

List of Tables

1 OLS model predicting mortality outcome missingness, all participants (n=301) . . . 4
2 OLS model predicting mortality outcome missingness, randomization phase (n=63) . 5
3 OLS model predicting mortality outcome missingness, treatment preference phase (n=238) 5
4 Participant characteristics in randomization and treatment choice phases 6
5 Mortality after prison release, randomization phase (n=61) 7
6 Mortality after prison release, treatment preference phase (n=230) 7
7 Causes of death 9
8 Cox proportional hazards models of survival, randomization phase 10
9 Cox proportional hazards models of survival, treatment preference phase 11

10 Cox proportional hazards models of survival, maximum observation time truncated to one year. 16
11 Cox proportional hazards models of survival, adding variable for time since study start. 17
12 Cox proportional hazards models of survival; as-treated: receipt of at least one methadone dose in 30 days before release. 18
13 Cox proportional hazards models of survival; as-treated: receipt of at least 60mg methadone dose before release. 19

1 Description of covariates

Baseline covariates used in survival analysis included a range of demographic, substance use, criminal justice and clinical variables. Demographic variables included age in years, education level completed (0=None, 1=Primary, 2=Form 1-Form 3, 3=Form 4-5, 4=Holding SPM, 5=Holding STPM/Diploma, 6=Degree/Master/PhD), race (categorized as Malay and non-Malay), marital status (categorized as married or not married, which included the categories of single and widowed). Substance use variables included whether individuals had used any alcohol, opioids, benzodiazepines or methamphetamine via any route of administration in the 30 days prior to incarceration, whether they had ever injected drugs, and the drug composite score from the Addiction Severity Index (scored from 0 to 100).[1] HIV-related and other clinical variables included whether individuals had ever received antiretroviral therapy prior to the time of entry into the study, baseline CD4 T-lymphocyte count (which was linearly rescaled in survival models so that a one-point increase represented a 100-cell increase), the HIV symptom index (a tally of physical and psychological symptoms, scored from 0 to 20),[2] and whether they had ever been diagnosed with tuberculosis prior to enrollment in the study.

References

1. McLellan AT, Kushner H, Metzger D, et al. The Fifth Edition of the Addiction Severity Index. J Subst Abuse Treat 1992; 9(3): 199-213.
2. Justice AC, Holmes W, Gifford AL, et al. Development and validation of a self-completed HIV symptom index. J Clin Epidemiol 2001; 54 Suppl 1: S77-90.

2 Testing the association between treatment group and outcome missingness

Allocation to methadone was not associated with outcome missingness in the full sample nor in the randomization or treatment choice phases of the study. For the full sample and the treatment choice phase, we fit unadjusted and covariate-adjusted models of outcome missingness. We failed to detect an association between outcome missingness and methadone in all models, as can be seen in appendix Tables 1 to 3, where the confidence intervals for the coefficients on methadone allocation overlap with zero.

Table 1: OLS model predicting mortality outcome missingness, all participants (n=301)

	Dependent variable:		
		Missing outcome	
	Model 1, β (95% CI)	Model 2, β (95% CI)	Model 3, β (95% CI)
Methadone	−0.006 (−0.052, 0.040)	−0.009 (−0.066, 0.048)	−0.004 (−0.062, 0.053)
Randomized allocation phase		−0.009 (−0.090, 0.073)	−0.009 (−0.094, 0.076)
Ever received antiretrovirals			−0.042 (−0.099, 0.014)
Age			0.003 (−0.001, 0.006)
CD4 T-cell count, baseline			−0.00002 (−0.0001, 0.0001)
Education level			−0.021* (−0.041, 0.0003)
Employed before incarceration*			−0.008 (−0.054, 0.037)
Sentence length, years			0.001 (−0.005, 0.006)
ASI Drug Composite Score			0.0002 (−0.002, 0.003)
Ever injected drugs			−0.004 (−0.107, 0.098)
HIV Symptom Index			−0.002 (−0.008, 0.004)
Lifetime tuberculosis diagnosis			0.016 (−0.035, 0.067)
Benzodiazpine use*			0.010 (−0.057, 0.078)
Methamphetamine use*			0.005 (−0.039, 0.049)
Alcohol use*			−0.051* (−0.108, 0.005)
Opioid use*			0.034 (−0.051, 0.120)
Malay ethnicity			−0.024 (−0.076, 0.028)
Married			0.035 (−0.032, 0.102)
Methadone*allocation phase		0.008 (−0.098, 0.114)	−0.002 (−0.110, 0.106)
Constant	0.038* (−0.002, 0.077)	0.041 (−0.010, 0.091)	−0.001 (−0.222, 0.219)
Observations	301	301	301
R^2	0.0002	0.0004	0.058
F Statistic	0.062 (df = 1; 299)	0.035 (df = 3; 297)	0.903 (df = 19; 281)
Note:			*p<0.1; **p<0.05; ***p<0.01
			*Assessed 30 days pre-incarceration.

Table 2: OLS model predicting mortality outcome missingness, randomization phase (n=63)

	Dependent variable:
	Missing outcome<br>β (95% CI)
Methadone	−0.001 (−0.089, 0.087)
Constant	0.032 (−0.030, 0.095)
Observations	63
R^2	0.00001
F Statistic	0.001 (df = 1; 61)

Table 3: OLS model predicting mortality outcome missingness, treatment preference phase (n=238)

	Dependent variable:	
	Missing outcome	
	Model 1, β (95% CI)	Model 2, β (95% CI)
Methadone	−0.009 (−0.066, 0.048)	−0.010 (−0.069, 0.049)
Ever received antiretrovirals		−0.036 (−0.098, 0.026)
Age		0.001 (−0.003, 0.005)
CD4 T-cell count, baseline		−0.00004 (−0.0001, 0.00005)
Education level		−0.015 (−0.039, 0.010)
Employed before incarceration*		−0.008 (−0.060, 0.044)
Sentence length, years		0.001 (−0.005, 0.007)
ASI Drug Composite Score		−0.001 (−0.004, 0.002)
Ever injected drugs		0.004 (−0.114, 0.121)
HIV Symptom Index		−0.002 (−0.009, 0.005)
Lifetime tuberculosis diagnosis		0.007 (−0.050, 0.065)
Benzodiazpine use*		−0.022 (−0.100, 0.056)
Methamphetamine use*		0.005 (−0.046, 0.056)
Alcohol use*		−0.045 (−0.108, 0.019)
Opioid use*		0.036 (−0.064, 0.136)
Malay ethnicity		−0.032 (−0.091, 0.028)
Married		0.011 (−0.069, 0.091)
Constant	0.041 (−0.010, 0.091)	0.091 (−0.164, 0.346)
Observations	238	238
R^2	0.0004	0.049
F Statistic	0.098 (df = 1; 236)	0.667 (df = 17; 220)
Note:		*Assessed in 30 days pre-incarceration.

3 Differences in baseline characteristics between randomized and treatment choice phase participants

Participants in the randomization and treatment choice phases differed significantly on baseline characteristics (p=0.004, F-test). More participants in the treatment choice phase reported ever having received antiretroviral therapy (p=0.006) and being employed before incarceration (p=0.011). The distribution of the Addiction Severity Index's drug composite score was significantly different between phases (p=0.028).

	Randomization phase	Treatment choice phase	p-value
Ever received antiretrovirals	9.8%	23.0%	0.006
Age	38.5	39.1	0.928
CD4 T-cell count, baseline	400	457	0.296
Education level	2.34	2.27	0.848
Employed before incarceration*	49.2%	67.8%	0.011
Sentence length, years	2.1	3.0	0.064
ASI Drug Composite Score	26.5	25.1	0.028
Ever injected drugs	95.1%	95.2%	0.966
HIV Symptom Index	5.3	4.8	0.478
Lifetime tuberculosis diagnosis	19.7%	23.9%	0.471
Benzodiazpine use*	9.8%	12.2%	0.597
Methamphetamine use*	44.3%	58.3%	0.055
Alcohol use*	18.0%	19.6%	0.785
Opioid use*	91.8%	93.5%	0.669
Malay ethnicity	68.9%	73.9%	0.448
Married	14.8%	10.0%	0.343

Table 4: Participant characteristics in randomization and treatment choice phases. For continous variables, values are means with p-values from a t-test; for dichotomous variables, values are percentages with p-values from a KS test. *Assessed in 30 days pre-incarceration.

4 Mortality rates by study phase

	Time (PY)	Deaths	Deaths per 1000 PY (95% CI)
Methadone	105.4	10	94.9 (42 - 172.1)
No Methadone	120.2	12	99.8 (52.8 - 156.6)
Overall	225.6	22	97.5 (61.4 - 141.5)

Table 5: Mortality after prison release, randomization phase (n=61)

	Time (PY)	Deaths	Deaths per 1000 PY (95% CI)
Methadone	502.5	33	65.7 (44.7 - 89.5)
No Methadone	144.4	7	48.5 (16.4 - 91)
Overall	646.9	40	61.8 (43.8 - 82.1)

Table 6: Mortality after prison release, treatment preference phase (n=230)

5 Causes of death

Causes of death in the national death registry were not reported systematically. We broadly classified deaths as infectious (53/62) and non-infectious (9/62) based on available data. The most common reported causes of death were sepsis (27/62), pneumonia (10/62), and pulmonary tuberculosis (6/62).

Cause of Death	Classification	Intervention
CEREBRAL TOXOPLASMOSIS	Infectious	No Methadone
HIV, UNSPECIFIED	Infectious	Methadone
HIV, UNSPECIFIED	Infectious	Methadone
HIV, UNSPECIFIED	Infectious	No Methadone
HIV, UNSPECIFIED	Infectious	No Methadone
HIV, UNSPECIFIED	Infectious	No Methadone
MENINGITIS, TUBERCULOUS	Infectious	Methadone
MENINGITIS, UNSPECIFIED	Infectious	Methadone
OPPORTUNISTIC INFECTION, UNSPECIFIED	Infectious	No Methadone
PNEUMONIA, PCP	Infectious	Methadone
PNEUMONIA, UNSPECIFIED	Infectious	Methadone
PNEUMONIA, UNSPECIFIED	Infectious	Methadone
PNEUMONIA, UNSPECIFIED	Infectious	Methadone
PNEUMONIA, UNSPECIFIED	Infectious	Methadone
PNEUMONIA, UNSPECIFIED	Infectious	Methadone
PNEUMONIA, UNSPECIFIED	Infectious	Methadone
PNEUMONIA, UNSPECIFIED	Infectious	Methadone
PNEUMONIA, UNSPECIFIED	Infectious	Methadone
PNEUMONIA, UNSPECIFIED	Infectious	Methadone
PULMONARY TUBERCULOSIS	Infectious	Methadone
PULMONARY TUBERCULOSIS	Infectious	Methadone
PULMONARY TUBERCULOSIS	Infectious	Methadone
PULMONARY TUBERCULOSIS	Infectious	Methadone
PULMONARY TUBERCULOSIS	Infectious	No Methadone
PULMONARY TUBERCULOSIS	Infectious	No Methadone
SEPSIS	Infectious	Methadone
SEPSIS	Infectious	Methadone
SEPSIS	Infectious	Methadone
SEPSIS	Infectious	Methadone
SEPSIS	Infectious	Methadone
SEPSIS	Infectious	Methadone
SEPSIS	Infectious	Methadone
SEPSIS	Infectious	Methadone
SEPSIS	Infectious	No Methadone
SEPSIS	Infectious	No Methadone
SEPSIS	Infectious	No Methadone
SEPSIS	Infectious	No Methadone
SEPSIS	Infectious	No Methadone
SEPSIS SECONDARY TO BURN	Infectious	Methadone
SEPSIS SECONDARY TO CEREBRAL TOXOPLASMOSIS	Infectious	No Methadone
SEPSIS SECONDARY TO ENCEPHALITIS	Infectious	No Methadone
SEPSIS SECONDARY TO OPPORTUNISTIC INFECTION, UNSPECIFIED	Infectious	Methadone
SEPSIS SECONDARY TO PCP PNEUMONIA	Infectious	Methadone
SEPSIS SECONDARY TO PNEUMONIA	Infectious	Methadone
SEPSIS SECONDARY TO PNEUMONIA	Infectious	Methadone
SEPSIS SECONDARY TO PNEUMONIA	Infectious	Methadone
SEPSIS SECONDARY TO PNEUMONIA	Infectious	Methadone
SEPSIS SECONDARY TO PNEUMONIA	Infectious	Methadone
SEPSIS SECONDARY TO PNEUMONIA	Infectious	Methadone
SEPSIS SECONDARY TO PNEUMONIA	Infectious	Methadone
SEPSIS SECONDARY TO PNEUMONIA	Infectious	No Methadone
SEPSIS SECONDARY TO PULMONARY TUBERCULSIS	Infectious	No Methadone
TUBERCULOSIS, DISSEMINATED	Infectious	No Methadone
ACUTE CORONARY SYNDROME	Not Infectious	Methadone
ACUTE CORONARY SYNDROME	Not Infectious	Methadone
GASTROINTESTINAL BLEED	Not Infectious	Methadone
LIVER CANCER, UNSPECIFIED	Not Infectious	No Methadone
LIVER FAILURE	Not Infectious	Methadone
RESPIRATORY DISTRESS, UNSPECIFIED	Not Infectious	Methadone
STEVEN JOHNSON SYNDROME	Not Infectious	No Methadone
STROKE	Not Infectious	Methadone
TRAUMA SECONDARY TO MOTOR VEHICLE ACCIDENT	Not Infectious	Methadone

Table 7: Causes of death

6 Treatment effect estimation by study phase

As with the full sample, our estimates of the impact of methadone on survival were consistent will a null effect in subanalyses in the randomized and treatment choice phases.

Table 8: Cox proportional hazards models of survival, randomization phase

	Dependent variable:		
	Death		
	Model 1, HR (95% CI)	Model 2, HR (95% CI)	Model 3, HR (95% CI)
Methadone	0.94 (0.41, 2.16)	0.84 (0.36, 1.93)	0.72 (0.20, 2.64)
Ever received antiretrovirals			3.50 (0.69, 17.82)
Age			0.92 (0.83, 1.02)
CD4 T-cell count, baseline+		0.86* (0.72, 1.02)	0.97 (0.74, 1.27)
Education level			1.08 (0.65, 1.79)
Employed before incarceration			2.04 (0.67, 6.20)
Sentence length, years			0.74 (0.48, 1.14)
ASI Drug Composite Score			1.03 (0.97, 1.08)
Ever injected drugs			1.33 (0.18, 9.64)
HIV Symptom Index			0.95 (0.79, 1.16)
Lifetime tuberculosis diagnosis			0.56 (0.16, 2.03)
Benzodiazpine use*			0.47 (0.05, 4.71)
Methamphetamine use*			2.31 (0.70, 7.66)
Alcohol use*			5.67 (0.52, 62.23)
Opioid use*			0.40 (0.05, 3.20)
Malay ethnicity			0.71 (0.09, 5.70)
Married			3.42 (0.67, 17.44)
Observations	61	61	61

Note: *Assessed in 30 days pre-incarceration. +Represents 100-cell/mm3 change.

Table 9: Cox proportional hazards models of survival, treatment preference phase

	Dependent variable:		
		Death	
	Model 1, HR (95% CI)	Model 2, HR (95% CI)	Model 3, HR (95% CI)
Methadone	1.34 (0.59, 3.06)	1.25 (0.54, 2.87)	1.19 (0.48, 2.97)
Ever received antiretrovirals			0.61 (0.22, 1.70)
Age			0.99 (0.93, 1.07)
CD4 T-cell count, baseline+		0.91 (0.81, 1.02)	0.82 (0.69, 0.98)
Education level			1.37 (0.93, 2.02)
Employed before incarceration			0.40 (0.20, 0.77)
Sentence length, years			1.07 (1.00, 1.14)
ASI Drug Composite Score			0.98 (0.94, 1.02)
Ever injected drugs			0.89 (0.22, 3.66)
HIV Symptom Index			0.97 (0.88, 1.07)
Lifetime tuberculosis diagnosis			0.58 (0.23, 1.49)
Benzodiazpine use*			2.39 (0.84, 6.84)
Methamphetamine use*			0.67 (0.28, 1.63)
Alcohol use*			1.73 (0.80, 3.73)
Opioid use*			6.85 (0.57, 81.99)
Malay ethnicity			0.47 (0.18, 1.23)
Married			1.89 (0.66, 5.43)
Observations	230	230	230
Note:	*Assessed in 30 days pre-incarceration. +Represents 100-cell/mm3 change.		

Figure 1: Unadjusted Kaplan-Meier survival curve of participants treated with within-prison methadone and controls in the **randomization phase.**

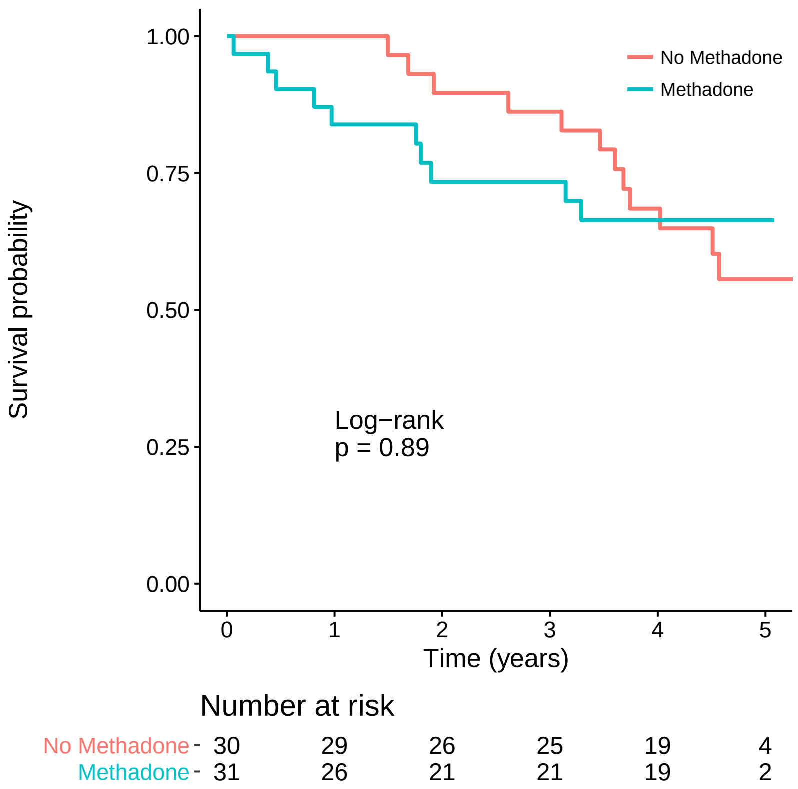

Figure 2: Adjusted Kaplan-Meier survival curve of participants treated with within-prison methadone and controls in the **randomization phase.** Curves are adjusted for baseline covariates using inverse probability weighting.

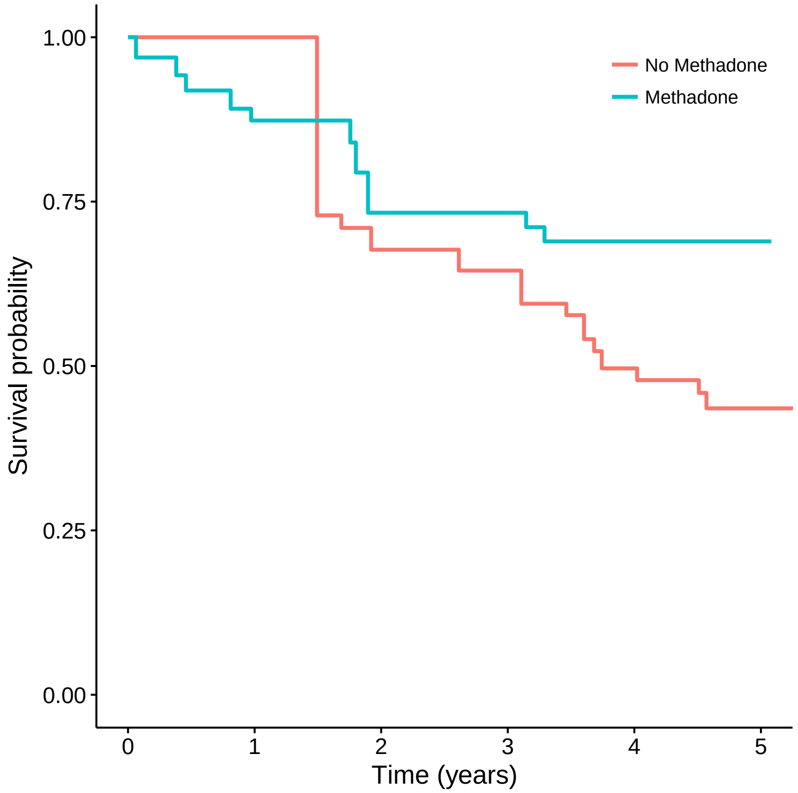

Figure 3: Unadjusted Kaplan-Meier survival curves of participants treated with within-prison methadone and controls in the **treatment choice phase.**

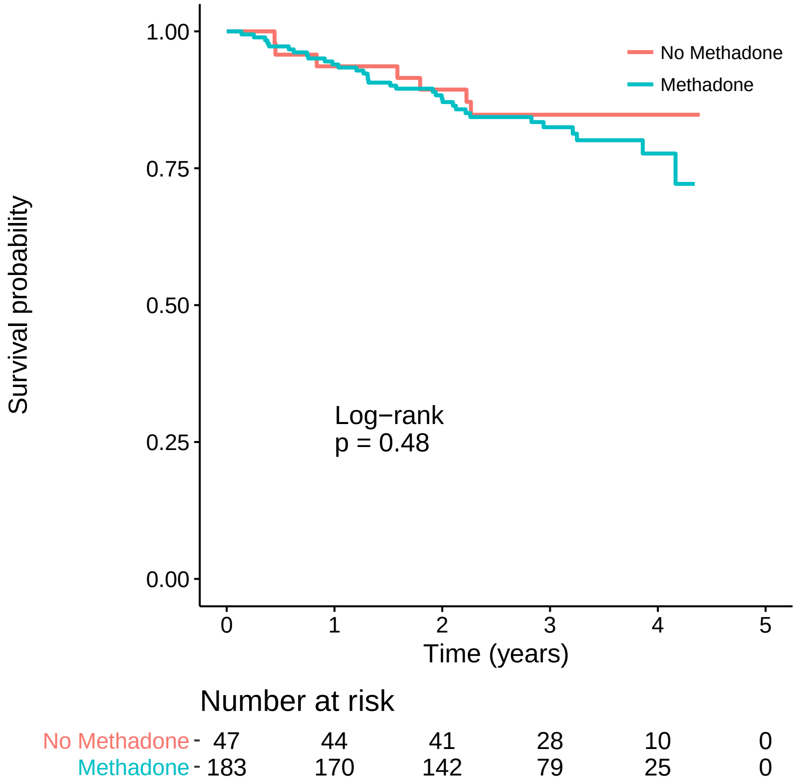

Figure 4: Adjusted Kaplan-Meier survival curves of participants treated with within-prison methadone and controls in the **treatment choice phase.** Curves are adjusted for baseline covariates using inverse probability weighting.

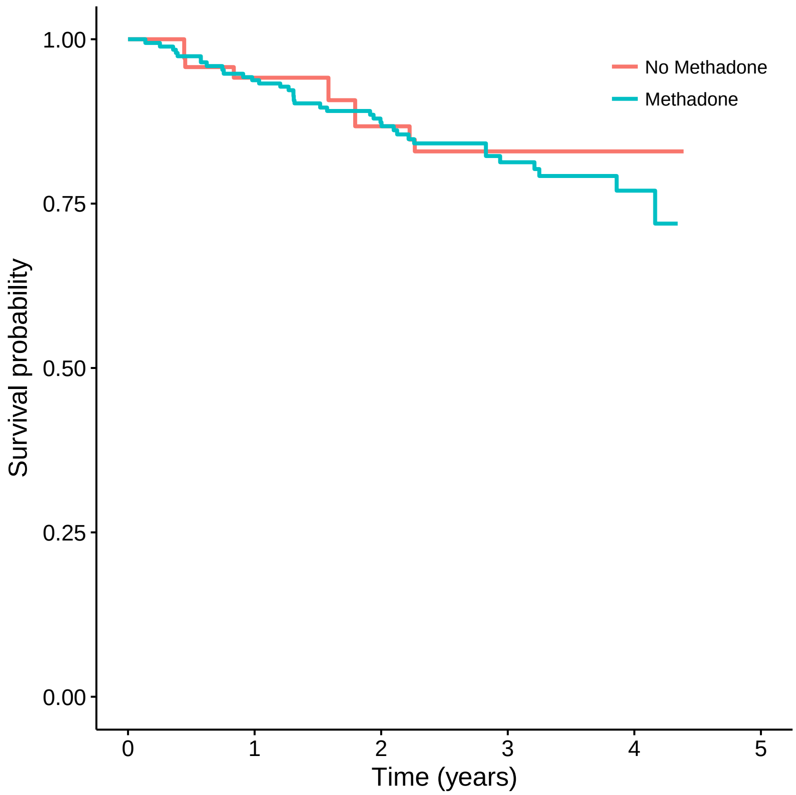

7 Sensitivity analyses

Table 10: Cox proportional hazards models of survival, maximum observation time truncated to one year.

	Dependent variable:		
	Death		
	Model 1, HR (95% CI)	Model 2, HR (95% CI)	Model 3, HR (95% CI)
Methadone	1.24 (0.54, 2.82)	1.17 (0.51, 2.66)	1.07 (0.46, 2.49)
Randomized allocation phase	2.98 (1.20, 7.41)	2.88 (1.17, 7.13)	2.59 (0.98, 6.82)
Ever received antiretrovirals			0.71 (0.31, 1.63)
Age			0.98 (0.93, 1.03)
CD4 T-cell count, baseline+		0.90 (0.82, 1.00)	0.85 (0.74, 0.97)
Education level			1.30 (1.00, 1.68)
Employed before incarceration			0.63 (0.36, 1.10)
Sentence length, years			1.05 (0.98, 1.12)
ASI Drug Composite Score			0.99 (0.96, 1.02)
Ever injected drugs			1.03 (0.36, 2.98)
HIV Symptom Index			0.97 (0.90, 1.04)
Lifetime tuberculosis diagnosis			0.57 (0.27, 1.22)
Benzodiazpine use*			1.73 (0.79, 3.79)
Methamphetamine use*			0.81 (0.43, 1.55)
Alcohol use*			2.01 (1.05, 3.86)
Opioid use*			2.03 (0.55, 7.50)
Malay ethnicity			0.62 (0.30, 1.26)
Married			2.33 (1.10, 4.92)
Methadone*allocation phase	0.67 (0.21, 2.14)	0.65 (0.21, 2.07)	0.57 (0.17, 1.85)
Observations	291	291	291
Score (Logrank) Test	10.87 (df = 3)	14.12 (df = 4)	38.84 (df = 19)
Note:	*Assessed in 30 days pre-incarceration. +Represents 100-cell/mm3 change.		

Table 11: Cox proportional hazards models of survival, adding variable for time since study start.

	Dependent variable:	
	Death	
	Model 1, HR (95% CI)	Model 2, HR (95% CI)
Methadone	1.24 (0.54, 2.87)	1.17 (0.48, 2.84)
Randomized allocation phase	1.98 (0.72, 5.45)	1.78 (0.51, 6.16)
CD4 T-cell count, baseline+	0.90 (0.82, 0.99)	0.84 (0.73, 0.96)
Education level		1.35 (1.04, 1.75)
Employed before incarceration		0.59 (0.33, 1.05)
Sentence length, years		1.05 (0.99, 1.12)
ASI Drug Composite Score		0.98 (0.95, 1.02)
Ever injected drugs		1.04 (0.38, 2.84)
HIV Symptom Index		0.97 (0.90, 1.05)
Lifetime tuberculosis diagnosis		0.56 (0.25, 1.28)
Benzodiazpine use*		1.83 (0.82, 4.09)
Methamphetamine use*		0.83 (0.43, 1.59)
Alcohol use*		2.01 (1.04, 3.87)
Opioid use*		2.41 (0.57, 10.28)
Malay ethnicity		0.61 (0.29, 1.27)
Married		2.01 (0.89, 4.52)
Months since study start	1.00 (0.97, 1.04)	1.00 (0.97, 1.04)
Ever received antiretrovirals		0.72 (0.31, 1.66)
Age		0.98 (0.93, 1.04)
Methadone*allocation phase	0.70 (0.22, 2.25)	0.66 (0.19, 2.33)
Observations	291	291
Score (Logrank) Test	6.54 (df = 5)	31.90 (df = 20)
Note:	*Assessed in 30 days pre-incarceration. +Represents 100-cell/mm3 change.	

8 As-treated analyses

Two "as-treated" analyses were conducted. The first defined treatment as receipt of at least one dose of methadone in the 30 days before release (n=203 treated, n=77 control, n=10 excluded for not receiving at least one dose of methadone in 30 days before release; analytic sample n=281). The second defined treatment as receipt of at least a 60 mg daily methadone dose before release (n=121 treated, n=77 control, n=30 excluded for missing pre-release dosing data, n=63 excluded for being allocated to methadone and failing to reach a 60 mg daily dose; analytic sample n=198). Estimated treatment effects from both as-treated analyses were consistent with a null effect.

Table 12: Cox proportional hazards models of survival; as-treated: receipt of at least one methadone dose in 30 days before release.

	Dependent variable:		
	Death		
	Model 1, HR (95% CI)	Model 2, HR (95% CI)	Model 3, HR (95% CI)
Methadone	1.40 (0.61, 3.20)	1.30 (0.57, 2.98)	1.25 (0.52, 2.99)
Randomized allocation phase	1.99 (0.81, 4.87)	1.85 (0.76, 4.52)	1.64 (0.58, 4.64)
Ever received antiretrovirals			0.72 (0.31, 1.66)
Age			0.99 (0.94, 1.04)
CD4 T-cell count, baseline+		0.90 (0.82, 1.00)	0.84 (0.73, 0.97)
Education level			1.32 (1.01, 1.71)
Employed before incarceration			0.61 (0.34, 1.08)
Sentence length, years			1.04 (0.97, 1.12)
ASI Drug Composite Score			0.98 (0.95, 1.02)
Ever injected drugs			0.91 (0.35, 2.37)
HIV Symptom Index			0.97 (0.90, 1.05)
Lifetime tuberculosis diagnosis			0.51 (0.21, 1.23)
Benzodiazpine use*			1.79 (0.79, 4.08)
Methamphetamine use*			0.82 (0.43, 1.60)
Alcohol use*			2.11 (1.09, 4.07)
Opioid use*			1.93 (0.49, 7.53)
Malay ethnicity			0.61 (0.30, 1.27)
Married			1.83 (0.85, 3.93)
Methadone*allocation phase	0.61 (0.18, 2.02)	0.60 (0.18, 1.98)	0.58 (0.16, 2.08)
Observations	281	281	281
Score (Logrank) Test	2.27 (df = 3)	5.67 (df = 4)	29.75 (df = 19)
Note:	*Assessed in 30 days pre-incarceration. +Represents 100-cell/mm3 change.		

Table 13: Cox proportional hazards models of survival; as-treated: receipt of at least 60mg methadone dose before release.

	Dependent variable:		
	Death		
	Model 1, HR (95% CI)	Model 2, HR (95% CI)	Model 3, HR (95% CI)
Methadone	1.25 (0.51, 3.09)	1.11 (0.45, 2.76)	1.01 (0.39, 2.60)
Randomized allocation phase	1.98 (0.81, 4.82)	1.82 (0.75, 4.40)	1.78 (0.60, 5.29)
Ever received antiretrovirals			0.77 (0.30, 1.97)
Age			0.97 (0.91, 1.04)
CD4 T-cell count, baseline+		0.87 (0.77, 0.98)	0.80 (0.67, 0.96)
Education level			1.40 (1.04, 1.89)
Employed before incarceration			0.81 (0.40, 1.63)
Sentence length, years			1.00 (0.89, 1.12)
ASI Drug Composite Score			0.99 (0.95, 1.03)
Ever injected drugs			0.91 (0.36, 2.31)
HIV Symptom Index			0.95 (0.86, 1.04)
Lifetime tuberculosis diagnosis			0.59 (0.21, 1.65)
Benzodiazpine use*			1.54 (0.55, 4.30)
Methamphetamine use*			1.01 (0.44, 2.36)
Alcohol use*			2.43 (1.16, 5.11)
Opioid use*			2.24 (0.39, 12.93)
Malay ethnicity			0.56 (0.23, 1.34)
Married			2.69 (1.06, 6.79)
Methadone*allocation phase	0.89 (0.25, 3.12)	0.91 (0.26, 3.16)	0.69 (0.18, 2.69)
Observations	198	198	198
Score (Logrank) Test	3.71 (df = 3)	7.90 (df = 4)	30.32 (df = 19)
Note:	*Assessed in 30 days pre-incarceration. +Represents 100-cell/mm3 change.		
